# Shared genes related to aggression, rather than chemical communication, are associated with reproductive dominance in paper wasps (*Polistes metricus*)

**DOI:** 10.1186/1471-2164-15-75

**Published:** 2014-01-28

**Authors:** Amy L Toth, John F Tooker, Srihari Radhakrishnan, Robert Minard, Michael T Henshaw, Christina M Grozinger

**Affiliations:** 1 Department of Ecology, Evolution, and Organismal Biology, Iowa State University, Ames, IA 50011, USA; 2 Department of Entomology, Iowa State University, Ames, IA, USA; 3 Department of Entomology, Center for Pollinator Research, Center for Chemical Ecology, The Pennsylvania State University, University Park, PA, USA; 4 Proteomics and Mass Spectrometry Core Facility, Huck Institutes for the Life Sciences, The Pennsylvania State University, University Park, PA, USA; 5 Department of Biology, Grand Valley State University, Allendale, MI, USA

**Keywords:** Wasps, Social behavior, Genomics, Aggression, Pheromones, Chemical communication

## Abstract

**Background:**

In social groups, dominant individuals may socially inhibit reproduction of subordinates using aggressive interactions or, in the case of highly eusocial insects, pheromonal communication. It has been hypothesized these two modes of reproductive inhibition utilize conserved pathways. Here, we use a comparative framework to investigate the chemical and genomic underpinnings of reproductive dominance in the primitively eusocial wasp *Polistes metricus.* Our goals were to first characterize transcriptomic and chemical correlates of reproductive dominance and second, to test whether dominance-associated mechanisms in paper wasps overlapped with aggression or pheromone-related gene expression patterns in other species. To explore whether conserved molecular pathways relate to dominance, we compared wasp transcriptomic data to previous studies of gene expression associated with pheromonal communication and queen-worker differences in honey bees, and aggressive behavior in bees, *Drosophila,* and mice.

**Results:**

By examining dominant and subordinate females from queen and worker castes in early and late season colonies, we found that cuticular hydrocarbon profiles and genome-wide patterns of brain gene expression were primarily associated with season/social environment rather than dominance status. In contrast, gene expression patterns in the ovaries were associated primarily with caste and ovary activation. Comparative analyses suggest genes identified as differentially expressed in wasp brains are not related to queen pheromonal communication or caste in bees, but were significantly more likely to be associated with aggression in other insects (bees, flies), and even a mammal (mice).

**Conclusions:**

This study provides the first comprehensive chemical and molecular analysis of reproductive dominance in paper wasps. We found little evidence for a chemical basis for reproductive dominance in *P. metricus*, and our transcriptomic analyses suggest that different pathways regulate dominance in paper wasps and pheromone response in bees. Furthermore, there was a substantial impact of season/social environment on gene expression patterns, indicating the important role of external cues in shaping the molecular processes regulating behavior. Interestingly, genes associated with dominance in wasps were also associated with aggressive behavior in bees, solitary insects and mammals. Thus, genes involved in social regulation of reproduction in *Polistes* may have conserved functions associated with aggression in insects and other taxa.

## Background

In many animal species, social interactions with conspecifics can profoundly influence individual physiology and behavior, including reproduction [1]. Eusocial insect societies represent an extreme case in which colonies consist of one or a small number of reproductively active queens or kings and tens to millions of sterile workers. In some species, direct physical aggression establishes reproductive dominance, while other species use chemical signaling via pheromones to establish dominance hierarchies [2]. It has been hypothesized that such chemical communication systems evolved from an ancestral state in which aggressive dominance interactions inhibited reproduction [3,4]. Comparative studies of the genomic mechanisms mediating reproductive dominance through aggression or chemical signaling can determine if these modes of communication share common genetic underpinnings.

Comparisons of the genomic mechanisms underlying social regulation of reproductive dominance in primitively eusocial *Polistes* wasps and advanced eusocial *Apis* honey bees provide an excellent framework with which to study the evolution of social inhibition of worker reproduction [5]. Eusocial behavior evolved separately in bee and wasp lineages [6], and thus, any shared mechanisms for sociality and reproduction between honey bees and paper wasps may represent deeply conserved elements that could be employed in multiple insect lineages [5]. On one extreme, *Polistes* wasps use physical aggression to initially establish dominance hierarchies, and then transition to ritualized behaviors, possibly using chemically based recognition to maintain these hierarchies [7]. On the other extreme, honey bee queens use pheromones to establish reproductive dominance, and physical aggression by queens towards workers is not observed. The effects of honey bee queen pheromones on worker physiology, behavior, and gene expression patterns have been extensively characterized [8-10]. Conversely, gene expression associated with dominance status in *Polistes* has not been previously studied, but such work is now possible with the development of microarrays to monitor genome wide expression patterns in *Polistes metricus* wasps [11]. The goal of this study was to compare dominance-related gene expression in *Polistes* to aggression- and pheromone-related gene expression patterns in honey bees and other species, allowing us to test whether reproductive dominance in *Polistes* is associated with genes with known links to aggression, pheromonal regulation, or both.

The presence of distinct groups of females on *Polistes* colonies exhibiting a range of caste, dominance, and reproductive states [12] provides an excellent opportunity for dissecting the mechanistic bases of reproductive dominance. The fact that dominance hierarchies occur in different stages of colony development also allows us to examine the importance of the colony environment, which has proven to be very important in recent studies in other social insects [13]. In temperate species of *Polistes*, one or a few sister females found annual colonies in the spring “founding phase” [14]. If multiple females are present, these foundresses form a dominance hierarchy with dominant foundresses (with large ovaries) taking over egg-laying and subordinate foundresses (with small ovaries) taking over foraging and provisioning of larvae [15]. During the “worker phase”, foundress-reared brood emerge as adult females, which typically become workers [12]. At this time, the dominant foundress increases egg-laying and is called the queen, and subordinate foundresses either die or are forced from the nest [16,17]. Linear dominance hierarchy among the females characterizes worker phase nests, with the queen as the alpha, or most dominant, individual [15]. Among workers, both dominant workers (with partially developed ovaries) and subordinate workers (with mostly undeveloped ovaries) are socially inhibited from reproducing, but subordinates even more so due to receiving more aggressive contacts and engaging in energetically demanding foraging behavior [18-20].

The initial position of a *Polistes* individual in a dominance hierarchy is established within a few minutes *via* intense aggressive interactions with other females, including biting, grappling, and attempted stinging [21]. Since behavioral dominance is established rapidly, it is not likely to involve large-scale changes in gene expression or physiology, although prior physiological and hormonal state influences performance in dominance contests [22-25]. Subsequently, repeated, more ritualized dominance interactions maintain physiological, or reproductive, dominance [26]. Dominance hierarchy maintenance requires chemical and/or visual individual recognition between wasps [27]. Large physiological changes in ovary activation, juvenile hormone, and ecdysteroid titers accompany this longer-term reproductive dominance [28-30]. Here, we focus on reproductive (physiological) dominance rather than behavioral dominance, as this form of dominance is longer-term and thus more likely to be manifest at the level of gene expression.

This study focuses on transcriptomic and chemical correlates of reproductive dominance, both within and between the different female castes in *Polistes metricus* wasps. First, we examined chemical profiles in subordinate and dominant *Polistes* workers, nest-founding females (dominant and subordinate co-foundresses), and queens, to determine if there were chemical correlates of caste and reproductive dominance, as suggested by previous studies with other species of *Polistes*[31,32], which could potentially function as chemical signals or cues to establish or maintain reproductive dominance hierarchies. Next, we examined the gene expression profiles in the brains and ovaries of these five groups of wasps, to explore transcriptomic correlates of caste and reproductive dominance. The goals of the transcriptomic study were two-fold: first, to provide new baseline data on patterns of gene expression associated with dominance to identify candidate genes for future studies, and second, to conduct a comparative genomic analysis by quantitatively comparing wasp dominance-associated gene expression patterns to gene expression data in other species. We hypothesized that paper wasp brain gene expression patterns would be related to gene expression associated with caste, exposure to queen pheromone and/or aggression in honey bee workers. In addition, we extended our comparative analysis to available data on aggression-related gene expression from two non-eusocial species, the fruit fly *Drosophila melanogaster* and the mouse *Mus musculus*. The results of these studies suggest reproductive hierarchies in primitively social species are associated with gene networks related to aggression in solitary species rather than pheromonal regulation in advanced eusocial species.

## Results

### Chemical analyses

We examined chemical profiles from dominant and subordinate co-foundresses (sampled during the founding phase of the colony) as well as queens and dominant and subordinate workers (sampled during the worker-producing phase of the colony). We identified four body areas as candidate carriers of dominance-related chemicals: 1) cuticular hydrocarbons, previously associated with dominance status in *P. dominula*[7,33,34]; 2) the mandibular glands, where queen pheromone is produced in honey bees [8], 3) Dufour’s glands because of their role in egg-marking in several social insect species [35], and 4) the sternal glands because of their potential importance to abdomen rubbing behavior which may accompany dominance interactions in *Polistes*[35]. Each of the four body regions examined had distinct chemical profiles, none of which were clearly related to dominance status. Data from the three glands are presented and discussed in the supplementary materials (Additional file 1: Supplemental Text, Figure S2).

**Figure 1:**
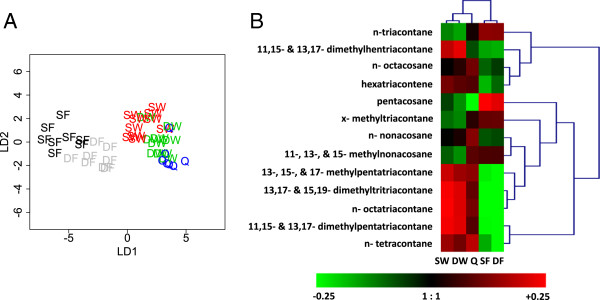
**Multivariate analyses of cuticular hydrocarbon data. A)** Linear discriminant analysis (LDA) of chemical profile data, showing graphs based on values of the two major linear discriminants, derived from quantities of compounds extracted from the cuticle from the five groups (DF = dominant foundress, SF = subordinate foundress, DW = dominant worker, SW = subordinate worker, Q = queen). **B)** Patterns of cuticular hydrocarbon abundance reveal a cluster of compounds related to season and/or social environment. Hierarchical clustering (represented by blue dendrograms) of mean values (log_10_ transformed) for 13 compounds with significant differences across the five female groups. The heatmap illustrates the fold difference in log_10_ levels of each compound compared to the overall mean for each compound (1:1), with higher levels in red and lower in green. Five compounds (bottom of the figure) show a similar pattern in which levels are lowest in foundresses and highest in workers and queens, reflecting differences in season and/or social environment.

From the cuticle, we identified 18 distinct hydrocarbons, a large proportion of which (13, or 72%) showed significant differences across the five groups of wasps (Table 1). Linear discriminant analysis (LDA) revealed a separation between the foundresses and the queen/worker groups (Figure 1A); this could possibly reflect differences in season or social environment. Using GC-MS, we identified at least 16 different hydrocarbons of varying chain lengths from 25-40 (Table 1). Thirteen of these compounds were previously identified as present on the cuticle of *Polistes metricus* (Table 1, [36]). Hierarchical clustering of the 13 compounds with significant differences among the treatment groups (Figure 1B) revealed a correlated cluster of five compounds in the range of 33-40 carbon chain length (13,17- and 15,19-dimethyltritriacontane, 13-,15- and 17-methylpentatriacontane, 11,15- and 13,19- dimethylpentatriacontane, n-octatriacontane, and n-tetracontane). All five compounds varied significantly among treatments (ANOVA, P < 0.05), and showed a similar pattern that reflected differences in season and/or social environment, with the highest levels in worker phase individuals (queens and workers) and lowest levels in founding phase individuals (foundresses).

**Table 1:** **Components of the cuticular hydrocarbons of *Polistes metricus***

**Putative ID**	**Retention time (min)**	**Carbon chain length**	**Molecular weight**	**p-value**
Pentacosane*	**27.6**	**25**	**352**	**0.004**
n-octacosane*	**34.6**	**28**	**394**	**0.006**
n-nonacosane*	**37.0**	**29**	**408**	**0.006**
11-, 13-, & 15-methylnonacosane*	**37.6**	**30**	**422**	**0.03**
n-triacontane*	**39.5**	**30**	**422**	**0.007**
n-hentriacontane*	41.1	31	436	NS
**x-methyltriacontane (?)**	**41.8**	**31**	**436**	**0.04**
n-dotriacontane*	42.3	32	450	NS
11,15- and 13,17-dimethylhentriacontane*	**45.0**	**33**	**464**	**0.004**
11,15- and 13,17-dimethylhentriacontane (?) *	45.7	33	464	NS
11-, 13-, 15- and 17-methyltritriacontane*	46.1	34	478	NS
Hexatriacontene isomer (?)	47.4	36	504	NS
**Hexatriacontene**	**47.8**	**36**	**504**	**0.008**
13,17- and 15,19-dimethyltritriacontane *	**49.4**	**35**	**492**	**<0.001**
13-,15- and 17-methylpentatriacontane *	**49.9**	**36**	**506**	**<0.001**
11,15- and 13,17-dimethylpentatriacontane *	**52.8**	**37**	**520**	**0.002**
**n-octatriacontane**	**53.3**	**38**	**534**	**<0.001**
**n-tetracontane**	**56.7**	**40**	**562**	**0.03**

All identifications were high confidence (>90% similarity to library or standards mass spectra) except for those indicated with (?). Compounds that differed in ANOVA analysis across the five groups are highlighted in bold and patterns presented graphically in Figure 1. Compounds with *were also identified as present in *Polistes metricus* in [36].

### Brain gene expression

Using previously developed custom oligo microarrays for *P. metricus*[11], we examined brain gene expression patterns of eight individuals from each of the five groups of wasps (dominant and subordinate co-foundresses, queens, and dominant and subordinate workers). Of the 5500 transcripts represented on the arrays, 3367 were expressed above background levels in a sufficient number of arrays to be included in the analysis. 499 of these (14.8%) were differentially regulated across the five behavioral groups (FDR p-value <0.05).

**Figure 2:**
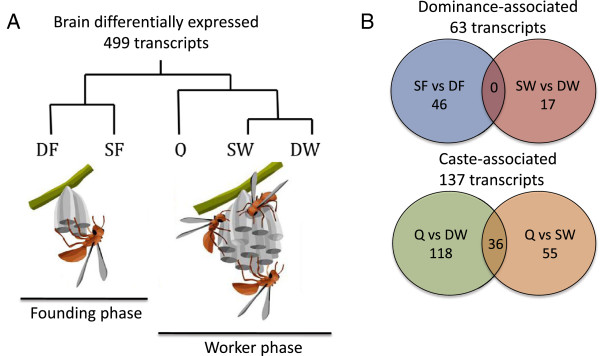
**Patterns of gene expression in brains of dominant and subordinate wasps.** A summary of the brain microarray data, for 499 differentially regulated transcripts across the five groups (DF = dominant foundress, SF = subordinate foundress, DW = dominant worker, SW = subordinate worker, Q = queen). **A)** Consensus clustering analysis (from both principal components analysis and hierarchical clustering) shows that many transcripts showed a pattern that corresponds to the social environment (founding phase or worker phase) and/or season. Wasp nest cartoons adapted from [37]. **B)** Venn diagrams summarizing the number of differentially regulated transcripts associated with either dominance status (top) or caste (bottom) and showing the overlaps between contrasts used to identify 'brain dominance-associated' transcripts (top) and 'brain caste-associated' transcripts (bottom).

Differentially regulated transcripts showed multiple distinct expression patterns across the five groups (multivariate analyses: Additional file 1: Figure S3). As in the case of the cuticular hydrocarbon profiles, there are clear differences associated with the two colony developmental phases. This effect of season/social environment on brain expression patterns was apparent in both hierarchical clustering based on a distance matrix of all possible contrasts (Figure 2A, Additional file 1: Figure S3C) and principal components analysis (PCA, Additional file 1: Figure S3B), where season/social environment accounted for 23% of the overall expression variation.

Post-hoc contrasts across the groups (FDR p-value < 0.05) revealed relatively few transcripts were associated with dominance status. We focused on two contrasts (dominant vs subordinate foundresses, and dominant vs subordinate workers) because these contrasts represented females that were interacting together on the same nest and were not confounded by comparisons across castes. There were 46 differentially regulated transcripts between dominant and subordinate foundresses and 17 differentially regulated transcripts between dominant workers and subordinate workers. There was no overlap across these two contrasts, suggesting again a potent influence of season or social environment in that different mechanisms appear to be associated with dominance in foundresses and workers (Figure 2B). The 63 transcripts showing differences between dominant and subordinate females (the union of the aforementioned two contrasts) are heretofore referred to as “brain dominance-associated” transcripts (Figure 2B, Additional file 2).

Similarly, to identify caste-associated genes, we examined overlapping sets of genes from queen vs worker post-hoc contrasts (FDR p-value < 0.05) and found a somewhat larger signal of differential expression. There were 118 differentially regulated transcripts between queens and dominant workers and 55 between queens and subordinate workers, 36 of which overlapped between the two contrasts (Figure 2B). The 137 transcripts showing differences between queens and workers (the union of the aforementioned two contrasts) are heretofore referred to as “brain caste-associated” transcripts (Figure 2B, Additional file 2).

We validated array expression data for one gene, *vitellogenin*, which was previously examined in queens and subordinate workers using quantitative real time PCR (qRT-PCR) [38]. Expression patterns uncovered with the array in the current study showed strikingly similar patterns to previous qRT-PCR data, with approximately 2-fold higher expression in queens compared to subordinate workers in both studies (Additional file 1: Figure S5).

### Ovary gene expression

Next, we examined ovary gene expression patterns of eight individuals from each of the five groups of wasps (dominant and subordinate co-foundresses, queens, and dominant and subordinate workers). Out of 5500 transcripts represented on the array, 3349 were expressed above background levels in a sufficient number of arrays to be included in the analysis. Of those, we found a large proportion (2302, or 68.7% of transcripts) were differentially regulated across the five groups (after correcting for multiple testing, false discovery rate p-value <0.01).

**Figure 3:**
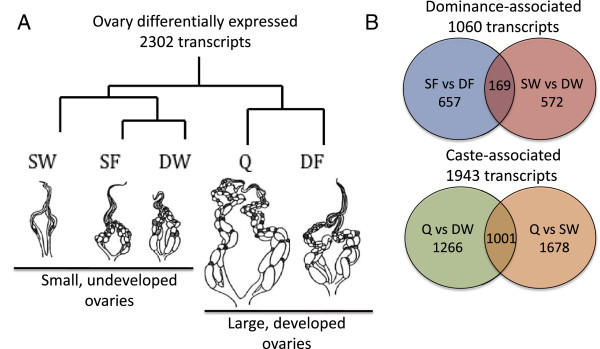
**Patterns of gene expression in ovaries of dominant and subordinate wasps.** A summary of the ovary microarray data, for 2302 differentially regulated transcripts across the five groups (DF = dominant foundress, SF = subordinate foundress, DW = dominant worker, SW = subordinate worker, Q = queen). **A)** Consensus clustering analysis (from both principal components analysis and hierarchical clustering) shows that many transcripts showed a pattern that corresponds to gross ovary activation state. Wasp ovary drawings adapted from [39]. **B)** Venn diagrams summarizing the number of differentially regulated transcripts associated with either dominance status (top) or caste (bottom) and showing the overlaps between contrasts used to identify 'ovary dominance-associated' transcripts (top) and 'ovary caste-associated' transcripts (bottom).

Again, there was a diversity of expression patterns across the five groups (multivariate analyses Additional file 1: Figure S4A, B, C). One of the most prevalent expression patterns (Figure 3A) reflects the gross level of ovary activation; queens and dominant foundresses had very high levels of ovary activation (score of 4) and were likely to be actively egg-laying, whereas the other three groups had very low ovary activation (scores of 1-2, and rarely 3, even in “dominant” workers) with no mature oocytes and were thus not actively egg-laying. This pattern was recovered both by hierarchical clustering by gene (Figure 3A, Additional file 1: Figure S4A) and PCA (Additional file 1: Figure S4B), in which ovary activation levels accounted for 41% of the variation.

Post-hoc contrasts across the groups (FDR p-value < 0.01) revealed a moderately large number of transcripts were associated with dominance status. There were 657 differentially regulated transcripts between dominant and subordinate foundresses and 572 differentially regulated transcripts between dominant workers and subordinate workers. There was an overlap of 169 transcripts across these two contrasts (Figure 3B), suggesting that there may be both shared and divergent mechanisms associated with ovary activation across the reproductive and worker castes. The 1060 transcripts showing differences between dominant and subordinate females (the union of the aforementioned two contrasts) are heretofore referred to as “ovary dominance-associated” transcripts (Additional file 3).

By examining overlapping sets of genes from queen vs worker post-hoc contrasts (FDR p-value < 0.01), we again found a larger signal of differential expression associated with caste differences. There were 1678 differentially regulated transcripts between queens and dominant workers and 1266 between queens and subordinate workers, 1001 of which overlapped between the two contrasts (Figure 3B). The 1943 transcripts showing differences between queens and workers (the union of the aforementioned two contrasts) heretofore referred to as “ovary caste-associated” transcripts (Additional file 3).

### Gene ontology (GO) analysis on dominance and caste-associated gene lists

Using DAVID [40], we tested to see which, if any, Gene Ontology categories (restricted to “Biological Process”) of genes were under or over-represented in our gene lists compared the background array. The 60 “brain dominance-associated” transcripts were represented by several small clusters of genes (shown in Table 2), none of which were significantly overrepresented in the gene lists relative to the background gene set on the array: eye development, reproduction, and cytoskeletal organization. For the “brain caste-associated” transcripts, only one GO category, oxidation reduction, was significantly overrepresented relative to the background on the array, and this was only significant with unadjusted p-values (Table 2). Other processes associated with, but not significantly overrepresented, in brain caste-associated genes included aging, synaptic transmission, and RNA processing.

**Table 2:** Summary of Gene Ontology (GO) Analysis of differentially expressed gene lists

**Cluster**	**Biological process of cluster**	**# genes**	**# Enriched subcategories**	**Example *Drosophila *** **homologs**
***Brain dominance-associated***				
C1	Compound eye development, photoreceptor cell differentiation	4	7/0	*COP9 complex homolog subunit 4, microtubule star, rasputin, scabrous*
C2	Cytoskeletal organization, actin filament organization	5	1/0	*Paramyosin, Transitional endoplasmic reticulum, upheld*
C3	Phagocytosis, vesicle mediated transport	4	0/0	*Beadex, alpha-coatomer protein*
C4	Reproduction, oogenesis	6	0/0	*COP9 complex homolog subunit , Glutamate dehydrogenase, quick-to-court*
C5	Nucleotide, ATP binding	8	0/0	*Hexokinase A, polyA-binding protein, rasputin*
C6	Zinc, ion, metal binding	7	0/0	*Nucleosome remodeling factor - 38kD, Sorbitol dehydrogenase-2, upheld*
***Brain caste-associated***				
C1	Oxidation reduction	12	8/0	*Ecdysone-induced protein 28/29kD, Glutathione peroxidase, Malate dehydrogenase, Sorbitol dehydrogenase-2*
C2	Aging, determination of adult life span	4	0/0	*Autophagy-specific gene 7, Excitatory amino acid transporter 1*
C3	Cell cycle process, microtubule-based process, cytoskeletal organization	8	0/0	*Eukaryotic initiation factor 4E, Helicase at 25E, Ribosomal protein L3, microtubule star, stubarista*
C4	Regulation of RNA metabolism	8	0/0	*Brahma associated protein 60kD, X box binding protein-1*
C5	RNA splicing, binding, processing	7	0/0	*Polyadenylate-binding protein 2, U2 small nuclear riboprotein auxiliary factor 50, hiiragi*
C6	Metamorphosis, morphogenesis, cell death	5	0/0	*Autophagy-specific gene 7, mastermind, scabrous*
C7	Synaptic transmission	5	0/0	*Glutamic acid decarboxylase , longitudinals lacking*
***Ovary dominance-associated***				
C1	Protein folding	18	0/0	*Cyclophilin 1, DnaJ-like-2, Heat shock protein cognate 4, T-complex Chaperonin 5*
C2	Proteolysis	52	1/0	*Serine protease inhibitor 4, amontillado, supernumerary limbs, Proteasome 29kD subunit*
C3	Mitotic spindle organization	36	7/0	*Replication Protein A 70, Ribosomal protein S4, short spindle 4, Dynein heavy chain 64C*
C4	Oxidative phosphorylation	16	0/0	*ATP synthase-beta, V-type proton ATPase subunit d 1, NADH:ubiquinone reductase 75kD subunit precursor*
C5	Regulation of cell projection, morphogenesis, differentiation	14	1/0	*Calcium/calmodulin-dependent protein kinase II, twinstar, short stop, capping protein alpha*
C6	Carboxylic and amino acid catabolic process	6	0/0	*Glutamate dehydrogenase, Probable maleylacetoacetate isomerase 2, sluggish A*
C7	Lipoprotein metabolism	4	0/0	*N-myristoyl transferase, Putative GPI-anchor transamidase, Rab escort protein*
***Ovary caste-associated***				
C1	Cytoskeletal organization, mitotic spindle organization	94	11/0	*Brahma associated protein 55kD, Dynamitin, Kinesin heavy chain, notch*
C2	Protein folding	29	1/1	*Calreticulin, Cyclophilin , Probable prefoldin subunit 4, Protein disulfide isomerase*
C3	Translation	61	3/0	*Elongation factor 1-gamma, Transcription factor IIB, Ribosomal protein S17*
C4	Cofactor metabolic/biosynthetic process	29	3/0	*Coenzyme Q biosynthesis protein 2, maroon-like, Succinate dehydrogenase B, Glutamate dehydrogenase*
C5	Generation of precursor metabolites and energy, oxidative phosphorylation	45	5/0	*Aconitase, Aldolase, Cytochrome c oxidase subunit Va, Pyruvate kinase*
C6	Proteolysis	83	8/0	*Diphenol oxidase A2, Insulin degrading metalloproteinase, Ubiquitin carrier protein, fizzy*
C7	Glucose and hexose metabolism	23	6/0	*Hexokinase A, Phosphoenolpyruvate carboxykinase, Phosphofructokinase*

Based on a GO analysis using *Drosophila* homologs**,** the top 6 or 7 clusters of GO terms corresponding to “Biological Process” are shown. Each cluster listed is accompanied by a description of the GO terms that make up the “Biological Process of Cluster”, “# genes” represented in each cluster, “# Enriched subcategories” which incidates GO subcategories that were significant within each cluster (counts refer to number of significant p-values, raw/FDR adjusted), and “Example *Drosophila* homologs” in each cluster.

In general, both “ovary dominance-associated” and “ovary caste-associated” genes showed functions related to protein folding, mitotic spindle organization, proteolysis, and metabolism (Table 2). For the “ovary caste-associated” list, there were a number of genes related to reproduction and ovary activation, though none of these were significantly enriched relative to the background. There was a cluster of 70 genes related to “reproductive process”, which included *Insulin Receptor Substrate, Sex lethal, Female sterile (2) ketel, Ecdysone induced protein 75B.* Another cluster of six genes was related to “oocyte fate determination”, and included *capping protein alpha, armadillo,* and *notch.*

### Comparative analysis

To begin to identify conserved pathways associated with caste and dominance, we tested for overlap between our complete lists of differentially regulated transcripts in brain (n = 502) and lists of differentially regulated transcripts from several studies in other species (Table 3).

**Table 3:** **Comparative analyses examining overlap in gene expression between *P. metricus*, *Apis*, *Drosophila*, and *Mus***

**Wasp list**	**Compared to X**	**Description of study**	**Citation**	**Sig. both**	**Sig. wasp only**	**Sig. X only**	**Sig. neither**	**p-value**
Brain DE	*Apis mellifera*	Queen vs sterile worker	[41]	77	129	354	498	0.322
Brain DE	*Apis mellifera*	Queen phero. response	[9]	58	339	384	2263	0.939
**Brain DE**	***Apis mellifera***	**Foragers vs nurses**	[42]	**106**	**99**	**392**	**487**	**<0.001**
Brain DE	*Apis mellifera*	Foragers vs nurses	[43]	58	340	254	1478	0.99
Brain DE	*Apis mellifera*	Aggression (composite)	[43]	85	312	512	2135	0.343
**Brain DE**	***Apis mellifera***	**Aggression (3 contexts)**	[43]	**5**	**8**	**392**	**2640**	**0.019**
**Brain Caste**	***Apis mellifera***	**Aggression (composite)**	[43]	**37**	**82**	**435**	**1575**	**0.022**
**Brain DE**	***D. melanogaster***	**Aggression**	[44]	**49**	**307**	**238**	**2247**	**0.0184**
**Brain DE**	***Mus musculus***	**Maternal aggression**	[45]	**27**	**200**	**77**	**960**	**0.033**
Brain DE	*Mus musculus*	Sleeping vs awake	[46]	68	159	323	714	0.692

Brain DE refers to the complete list of transcripts differentially expressed in the brain, “Brain Caste” refers to the subset of genes that are “brain caste-associated”, described in the main text. The number of transcripts overlapping (significant in both, or “Sig. both"), as well as non-overlapping transcripts (significant in wasp only, or ”Sig. wasp only”; significant in the other species, “Sig. X only”; and significant in neither study, “Sig. neither”) and two-tailed p-values from Fisher’s Exact Tests are shown. Lists of genes with significant overlaps are highlighted in bold and a complete list of overlapping transcripts are provided in Additional file 2.

We found no significant overlap between wasp brain differentially expressed transcripts and those differentially expressed in honey bees in association with caste differences [41] or response to queen pheromone [9]. We did find a significant overlap between wasp brain differentially expressed gene lists and those associated with differences in worker foraging behavior in honey bees in one study [42], but this result was not confirmed in comparisons with a second study on the same behavior [43].

We next investigated whether there was significant overlap with genes relating to aggressive behavior in honey bees. Alaux et al. [43] examined honey bee aggressive behavior in several contexts—as it relates to genotype (aggressive Africanized lineages compared to more docile European lineages), age (hive bees vs foragers), and response to alarm pheromone (which elicits attack and stinging behavior). We first focused on the subset of genes that were found to be differentially expressed in all three contexts in honey bees. We found a small, but significant overlap between this list of genes and the complete set of brain differentially expressed genes in wasps. This suggestive result, along with the small size of the gene lists being compared, led us to compile an expanded list of all genes related to honey bee aggressive behavior in any context (the union of the three contexts). Here we found no significant overlap with the wasp brain differentially expressed list; however, when we compared this expanded honey bee aggression list to the wasp "brain caste-associated" genes, we again found significant overlap (Table 3). There was no significant overlap when we compared to the 63 wasp "brain dominance-associated genes” (data not shown).

To further investigate the potential connection between genes related to dominance (our study) and aggression, we compared our complete wasp brain differentially expressed gene list to microarray studies identifying brain-expressed genes associated with aggression in *Drosophila melanogaster* fruit flies [44] and maternal aggression in mice [45]. In both cases, we found evidence of a relatively small, but statistically significant overlap (Table 3). As a control, we compared our study to another mouse study that used the same microarray to examine brain gene expression patterns associated with sleep [46]—no significant overlap was detected.

## Discussion

This study is the first comprehensive examination of chemical profiles and genome-wide expression patterns associated with reproductive dominance in a primitively eusocial species. Our analysis of cuticular hydrocarbons identified over a dozen compounds with potential links to the phase of the colony cycle (which encompasses season and social environment) in *P. metricus*. In addition, we provide new baseline data on transcriptomic correlates of reproductive dominance and caste in both brains and ovaries. Many genes showed expression patterns related to the social environment/season (founding phase vs worker phase, Figure 2), suggesting there could be major effects of social environment on brain gene expression in wasps. Thus, both the chemistry and brain transcriptome data show patterns strongly associated with the social environment, and highlight the fact that there are major differences in the social milieu between founding and worker phase colonies. These data agree with other recent studies suggesting the social environment as one of the most potent influences on gene expression patterns in ants [13]. Finally, our results indicate the brain expression patterns associated with reproductive dominance are (surprisingly) not conserved across wasps and honey bees, but rather that some genes associated with aggressive behaviors may have been co-opted to establish or maintain dominance hierarchies in *Polistes* wasps.

Previous studies have clearly demonstrated cuticular hydrocarbons change with female fertility in insects including *Polistes*, with some evidence for cuticular differences related to dominance status in foundresses/workers of other species of *Polistes* wasps [7,34,35,47,48]. We identified 13 cuticular hydrocarbons in *P. metricus* with significant differences across females. Several of these compounds have been identified on the cuticles of other insects, for example, in association with age in mosquitoes (pentacosane [49]), ovarian activation in social insects (dimethylpentatriacontane and dimethylhentriacontane [31], pentacosane, nonacosane, methylnonacosane, triacontane, and methyltriacontane [50]), and even dominance status in other species of *Polistes* (pentacosane, nonacosane, and methylnonacosane [32], methylpentatriacontane [51], and dimethylpentatriacontane [31]). However, in our study, none of these compounds were closely related to dominance status or levels of ovarian activation in *P. metricus*, but several showed associations with the time of collection (early vs late season) and/or social environment (foundress association vs queenright mature colony, Figure 1B). Further studies on these 13 compounds could provide additional insights into the role of cuticular hydrocarbons in response to the abiotic and social environment in *Polistes*.

Our microarray results suggest a relatively small subset of genes in the brain show patterns related to reproductive dominance status. There were several differentially expressed genes related to vision and eye development, which is intriguing because of the importance of visual communication in the genus *Polistes*, although *P. metricus* is not known to use visual cues for individual recognition [52,53]. We found no overlap between sets of differentially expressed genes between dominant and subordinate foundresses and dominant and subordinate workers, suggesting that distinct subsets of genes may be involved in the maintenance of dominance status in the founding and worker stages of colony development. This is consistent with known differences in the role of juvenile hormone (JH) in the development and maintenance of dominance status and ovarian activation in queens versus workers in *Polistes*. In foundresses, JH regulates behavioral and ovarian reproductive dominance [22,24,30]. In workers, JH has a dual function in that it affects both reproductive dominance [20] and age-related onset of foraging behavior [54,55], and JH action depends on the physiological condition (i.e. nutritional state) of the female [56,57].

A slightly larger subset of genes showed caste-associated expression differences in the brain. These genes had functions related to oxidation reduction, aging, and synaptic transmission, which could be linked to known differences in metabolism [58], lifespan [12], and learning abilities [59] between workers and queens. Previous studies in honey bees have also uncovered differences in the expression of genes related to aging (such as *telomerase*) and oxidation reduction [41]. We suggest candidate genes related to aging (*Autophagy-specific gene 7, Excitatory amino acid transporter 1*), eye development (*microtubule star, rasputin, scabrous*), and reproduction (*Glutamate dehydrogenase, quick-to-court*) may play an important role in establishing and maintaining adult caste differences in *Polistes.*

Our cross-species comparative analyses showed no significant overlap in sets of genes associated with dominance status in wasps and pheromonal regulation in honey bees. Thus, they do not support the hypothesis that pheromonal regulation of reproduction relies on the same molecular mechanisms as physical dominance in these two species. Furthermore, according to the ovarian and reproductive groundplan hypotheses [60-62], genes involved in reproduction have been co-opted to play a role in queen-worker caste differentiation and worker division of labor. However, in contrast to this theory, we find distinct brain gene expression patterns are associated with reproductive dominance hierarchies between dominant and subordinate co-foundresses and between dominant and subordinate workers, and dominance-associated genes differ between wasps and honey bees. Thus, there does not appear to be a conserved suite of genes regulating these processes in the brain. Interestingly, however, genes associated with dominance in *Polistes* significantly overlap with sets of genes associated with aggressive phenotypes in honey bees [43], *Drosophila*[44], and mice [45]. There was also some overlap with genes related to foraging in one [42] of two [43] previous honey bee studies. This overlap may reflect differences in aggressive behavior between honey bee foragers and non-foragers [43], or perhaps be explained by the fact that lower dominance status in wasps is typically associated with increased foraging behavior [12]. Overall, these data suggest that there may be a small number of genes with recurrent roles in aggressive behavior across diverse taxa. It is important to note, however, that the microarray only examined a subset of the genes in the paper wasp genome and was limited to transcripts showing significant homology to honey bee or other insect proteins. The role of novel genes or rapidly evolving genes in the regulation of dominance status in *Polistes* remains to be explored and is definitely worthy of further attention [63].

We found large differences in ovary gene expression, both associated with dominance status and with caste differences. Overall, many transcripts showed expression differences associated with gross differences in ovary size (Figure 3A). This pattern is reflected in the types of genes that were differentially expressed--there were numerous genes with functions related to cell division and proliferation, as well as production of nucleic acids and proteins. Thus, the large differences in ovary size across the groups (Figure 3A) are undoubtedly produced by changes in the regulation of genes related to egg production and maturation.

## Conclusions

In summary, experiments presented here provide a wealth of new data about the chemical and transcriptomic correlates of reproductive dominance in *Polistes* paper wasps, an important model system for studying dominance behavior and the evolution of sociality [74]. Several specific compounds and genes are excellent candidate for future studies of their causal role in establishing and maintaining dominance. Our data also highlight the importance of the season and/or social environment in gene expression and cuticular hydrocarbon production, and suggest there are distinct mechanisms responsible for communicating and maintaining dominance among foundresses, between queens and workers, and among workers. Comparisons with honey bees suggest that largely different sets of genes are associated with social regulation of reproduction in honey bees and paper wasps. This is not entirely surprising, considering bees and social vespids diverged between 100-150 million years ago [6], and that the form of social control of reproduction (chemical vs physical) differs greatly between the two species.

The most notable finding from our cross-species comparisons is that genes that are differentially expressed in brains of dominant and subordinate wasps are likely to be associated with aggression in other species, from honey bees, to flies, to mice. Our data suggest that in primitively social wasps, social regulation of reproduction may be regulated by genes with deeply conserved functions associated with aggression in solitary insects and other taxa. Thus, our data have begun to unravel the evolution of the mechanistic underpinnings of reproductive inhibition in workers, and that in some cases this may be built on fundamental elements of solitary behavior, such as aggression.

## Methods

### Wasps

We collected *Polistes metricus* adult females at four field sites in central Illinois (USA): Vermilion River Observatory (Danville, IL, +40°3′28″, -87°33′42″), Allerton Park (Monticello, IL, +40°0′25″, -88°38′58″), Lake of the Woods (Mahomet, IL, +40°12′6″, -88°22′38″), and Forest Glen (Westville, IL, +40°0′46″, -87°33′55″). We collected wasps from undisturbed nests located in wooden nest boxes or on the eaves of buildings between 5:30-7:00 am to ensure that all wasps were present on the nest and to control for circadian effects on gene expression. We collected 23 wasps during the founding phase between May 14-17, 2008 from nests with 2-3 females (10 nests with two and one nest with three females). We observed each nest >2 times in the 3 weeks prior to collection to verify the presence of multiple foundresses. Populations of *Polistes metricus* in Illinois generally have few nests that are multiply founded (~ 5%, A.L.T, personal observation). We collected 116 wasps during the "worker phase" between July 27- August 1, 2008 from 20 nests with at least 2 workers and no males; males are indicative of colonies producing non-worker reproductive females. To remove wasps from their nests, we anaesthetized them with CO_2_ gas for 30 sec, then immediately freeze-killed them on dry ice and stored them at -80°C for further analysis.

### Dissections and determinations of reproductive dominance rank

Each wasp was subjected to several dissections (Additional file 1: Figure S1). We removed legs on dry ice and stored them at -80°C for microsatellite analysis (see below). We noted wing wear (presence or absence) as an indicator of foraging experience [64]. We thawed gasters in RNA-later® (Qiagen, Valencia, CA), then dissected ovaries and scored ovary activation (1 = completely undeveloped, string-like ovarioles, 2 = slightly developed ovarioles with small bulges, 3 = partially developed ovarioles, with two or fewer fully developed oocytes, 4 = fully developed ovarioles, with three or more fully developed oocytes). We stored ovaries in RNA-later® at -80°C for RNA extractions. We dissected Dufour’s glands and sternal glands (located based on descriptions in [35]) from gasters and stored the glands in 200 μL diethyl ether. After dissection, we submerged each wasp’s gaster in 1 mL pentane for 10 minutes to extract cuticular hydrocarbons. We freeze-dried heads for 60 min at 300mTorr, dissected brains on dry ice, and stored brains at -80°C for RNA extractions. From the same heads we dissected mandibular glands (based on descriptions in [35]) and placed them in 200 μL diethyl ether.

### Choice of focal wasps for microarray and chemical analysis

To reduce variation due to differences in ovary activation, caste, and relatedness, we focused on a subset of collected wasps (n = 8 focal females per group) in each of five groups: DF = dominant foundress, SF = subordinate foundress, Q = queen, DW = dominant worker, SW = subordinate worker. Wasp relatedness was assessed using microsatellites (Additional file 1, complete data in Additional file 4); this information allowed us to identify and exclude wasps from nests with evidence of queen replacement, which can profoundly disrupt dominance hierarchies in *Polistes*[65]. We focused on foundress associations with exactly two females, inferred from microsatellites to be sisters (but due to limits on sample size we included two pairs of non-sisters). We chose pairs of workers from the same nest inferred to be sisters and the daughters of the resident queen. We used foundress pairs that had clear differences in reproductive dominance—the subordinate female had an ovary activation score of 1 and the dominant female a score of 4. For worker phase nests, we identified queens as females with ovary activation scores of 4 and high levels of wing wear (acquired during the founding phase, [64]). We chose dominant workers as females with no wing wear and ovary scores of 2 or 3, and subordinate workers as females with high levels of wing wear and ovary scores of 1.

### Chemical analysis

Methodology for analysis of the three glands (mandibular, Dufour's and sternal) are presented in Additional file 1. A 1 μL sub-sample of each cuticular extract was injected into an Agilent 6890 GC System using an Agilent HP-5MS column (30 m length × 0.25 mm diameter × 0.25um thickness) in splitless mode and a flame ionization detector. The temperature program was as follows: 150°C hold for 1 minute, ramp up 15°C/minute to 200°C, ramp up 7.5°C/minute to 300°C, hold 25 minutes. Data were quantitated using Agilent Chemstation and internal standards. Eluting compounds were identified by comparing retention times and spectra (GC-MS) with those of pure standards.

We chose a subset of representative cuticular samples for GC-MS analysis on a Waters GCT gc-tof-ms using a similar column in splitless mode at 1 mL/minute He flow. Injector 250°C; program: 50°C, hold 1 minute, 20°C/minute to 180°C, 3°C/minute to 320°C, hold 15 minutes. The identifications of 18 hydrocarbons from C25 (MW 352) to C40 (MW 562) were confirmed by either spectral comparison with the NIST MS Search 2.0 mass spectral library or by running standards.

### Microarrays

The *P. metricus* oligo microarrays [11] are comprised of 10,000 duplicate spots, representing 5000 different transcripts, corresponding to approximately 3248 different genes. We extracted each individual wasp’s brain and ovary RNA using a PicoPure (Evrogen, Moscow, Russia) kit. We assessed total RNA quantity with a NanoDrop (Thermo Scientific, Waltham, MA) and quality with an Agilent Bioanalyzer (Agilent, Santa Clara, CA). We then subjected each RNA sample to T7 amplification (Invitrogen, Grand Island, NY) and labeled each independently with both Cy3 and Cy5 dyes (Invitrogen, Grand Island, NY). We then hybridized amplified, labeled RNA to each microarray using previously described methods [11]. We employed a complete loop design with a dye swap, such that each individual wasp appeared on two arrays (8 wasps per group, 5 groups, 40 arrays, for both brain and ovary microarray studies). We hybridized arrays for approximately 18 hours at 42°C in a Maui mixer (BioMicro Systems, Salt Lake City, UT), then washed and immediately scanned them, with saturation tolerance set at 0.10%, using a GenePix scanner (Molecular Devices, Sunnyville, CA). We manually spot-checked all arrays to remove spots with irregular printing or dust on the array.

### Statistical analysis

For the chemical analysis, we calculated absolute concentrations of each compound for each individual sample using the external standard. These values were log-transformed and used for mixed model ANOVAs in R v. 2.15.3 (R Core Development Team 2008), with group (the five female types) as a fixed effect and colony as a random effect. Although the compounds on the cuticle are unlikely to be truly independent from each other, this analysis was useful as it allowed us to assess differences among groups for each compound separately. We conducted pairwise comparisons and adjusted p-values for multiple testing using a Bonferroni correction. We also used the same values for linear discriminant analysis (LDA) in R and hierarchical clustering analysis by compound (for significantly different compounds only, single linkage clustering method) in Genesis [49].

We used SAS to analyze microarray data as in [66]. We removed data from spots with intensity levels lower than the median background level of 175 and log transformed and normalized data across arrays using the Lowess method. We removed spots that were missing from >25% of the arrays. We used a mixed model ANOVA to test for differences in expression, with dye and array as random effects and group and spot as fixed effects. P-values were corrected for multiple testing using false discovery rate (FDR), likewise for all pairwise comparisons between the groups. We used an FDR p-value significance cutoff of 0.05 for brains and 0.01 for ovaries. A more stringent cutoff was used for ovaries because there were a considerably larger number of differentially expressed transcripts. The results were visualized using principal components analysis and hierarchical clustering in JMP (SAS Institute, Cary, NC). We generated a distance matrix based on the number of differentially regulated transcripts between each pairwise comparison of female type, which we used to conduct hierarchical clustering in R. We conducted Gene Ontology analysis in DAVID [40], using *Drosophila* best hits to the wasp transcripts (as in [11]), and using the full set of *Drosophila* best hits on the *P. metricus* array as a background list. We report results of overrepresentation tests, both raw and Benjamini adjusted p-values.

To conduct tests of cross-species overlap, for each species, we used tBLASTx of *P. metricus* transcripts against other species’ databases. Honey bee and *Drosophila* hits were used as described previously, with e-value cutoffs of 1 e^-5^[67]. For the mouse *Mus musculus*, we used BLAST2GO [68] against the Ensembl database and best tBLASTx hits were identified, with e-value cutoffs of 1e^-3^. If a *P. metricus* transcript did not have a hit meeting this cutoff, it was not used in further analyses. This resulted in lists of putative orthologs between *P. metricus* transcripts and each query species database. Using data in online repositories (GEO and MIAME), we identified the putative orthologs that were present on both test arrays being compared (*P. metricus* and either bee, fly, or mouse). We determined the overlap between gene lists and used two-tailed Fisher Exact tests to determine whether the number of genes that were shared in common between both species was significantly higher or lower than expected by chance, compared to a hypergeometric distribution.

We tested for overlap between transcripts differentially expressed in wasp brains and transcripts with expression patterns in the brains of honey bees that were significantly associated with: 1) caste differences in adult queens and workers [41]; 2) exposure of workers to queen mandibular pheromone verses a solvent control [9]; 3) behavioral state differences between foragers and nurses [42,69]; and 4) aggressive behavior in workers [43]. We also tested for overlap between lists of differentially regulated genes related to aggression in *Drosophila*[44] and the mouse *Mus musculus*[45]. Although we found numerous additional studies examining aggressive behavior in other species including a cichlid fish [70], chicken [71], human [72], and a songbird [73], we were unable to conduct meaningful overlap analyses because of the small number of genes that met both criteria of being differentially expressed and having homologous sequences in *P. metricus*.

### Availability of supporting data

All microarray data and details of the experiment were deposited in the Array Express database http://www.ebi.ac.uk/arrayexpress (ArrayExpress accession number E-MTAB-2190 for brain data and E-MTAB-2191 for ovary data) in accordance with MIAME (“Minimum information about a microarray experiment”) standards.

## Abbreviations

GC: Gas chromatography; GC-MS: Gas chromatography-mass spectrometry; RNA: Ribonucleic acid; LDA: Linear discriminant analysis; PCA: Principal components analysis; FDR: False discovery rate.

## Competing interests

The authors declare that they have no competing interests.

## Authors' contributions

ALT conceived of the experiment, conducted GC and microarray studies, analyzed the data, and wrote the paper. JFT conducted GC-MS analyses, analyzed GC-MS data, and helped to draft the manuscript. SR performed comparative analyses. RM conducted GC-MS analyses and analyzed GC-MS data. MTH performed microsatellite analyses and analyzed microsatellite data. CMG participated in the design and coordination of the study and helped to draft the manuscript. All authors read and approved the final manuscript.

## Supplementary Material

Additional file 1 Contains supplementary figures, tables, methods, results, and discussion to accompany the main text. Click here for file

Additional file 2 Contains a list of brain differentially expressed transcripts, including lists of which overlapped across the different cross-species comparisons. Click here for file

Additional file 3 Contains a list of ovary differentially expressed transcripts. Click here for file

Additional file 4 Contains microsatellite data. Click here for file
