# Supplementary material for: Shared genes related to aggression, rather than chemical communication, are associated with reproductive dominance in paper wasps (Polistes metricus)
Source: BMC Genomics. 2014 Jan 28;15:75. doi: 10.1186/1471-2164-15-75 (PMC3922164; doi:10.1186/1471-2164-15-75)
Supplement: Additional file 1 — Contains supplementary figures, tables, methods, results, and discussion to accompany the main text. [file 1471-2164-15-75-S1.docx]

**Additional File 1: Supplemental information**

**Supplemental Text**

*Microsatellites*

Relatedness has the potential to affect social interactions in *Polistes* [[1](#_ENREF_1)]. To avoid including wasps from nests that had experienced queen turnover events and to focus on sets of wasps with known relatedness, we used microsatellites to assess relatedness between wasps collected on the same nest. We genotyped foundresses, queens and workers at four microsatellite loci previously identified in *Polistes bellicosus* (Pbe128, Pbe203, Pbe205, Pbe269) and two loci previously identified in *Polistes dominula* (Pdom2, Pdom20) [[2](#_ENREF_2)] following previously published protocols [[3](#_ENREF_3)]*.* We estimated relatedness between the workers and the queens on each colony using the computer program *Relatedness* 5.0 [[4](#_ENREF_4)], and assessed the most likely pedigree relationship between workers and queens using the program *Kinship* 1.3 [[4](#_ENREF_4)].

Eight of 11 foundress pairs (72.7%) were highly related (consistent with being full sisters, as reported in [[5](#_ENREF_5)]). Eleven of 21 nests (52%) had workers that were highly related (consistent with a mother-daughter relationship) and 10 nests had lower relatedness, possibly due to colony usurpation or presence of multiple egg-laying females. Complete microsatellite results are in “Additional File 2”. For the remainder of our analyses, we focused on 16 nests (“Additional File 2”) with known relatedness structures, resulting in 8 founding phase pairs (6 of which were full sisters) and 8 worker phase trios (in all cases the workers were daughters of the queen).

*Chemical analyses*

We derivatized 20 μL samples from the mandibular glands, Dufour’s glands, and sternal glands overnight by adding 10 μL of BSTFA (N,O-Bis(trimethylsilyl)trifluoroacetamide, Sigma-Aldrich, St. Louis, MO). We then added 100μL of hexane with 100 ng/μL of a standard (octadecane for mandibular glands and Dufour’s glands, hexatriacontane for sternal glands). Temperature programs for each type of sample were as follows: 1) mandibular glands (35°C hold for 1 min, ramp up 5°C/min to 100°C, ramp up 10°C/min to 300°C, hold 5 min), 3) Dufour’s glands (100°C hold 1 min, 10°C/min to 300°C, hold 20 min), and 4) sternal glands (50°C hold 1 min, 5°C/min to 100°C, 10°C/min to 300°C, hold 15 min). Data were quantitated using Agilent Chemstation and internal standards. Data were analyzed exactly as described for the cuticular extracts in the main text.

Each of the three exocrine glands showed unique patterns of abundance of numerous unknown chemicals. Note that we did not identify these compounds using GC-MS, thus all results presented for glands refer to unidentified compounds only. For the mandibular glands, only two out of 40 compounds differed significantly across the five groups. A linear discriminant analysis (LDA) showed strikingly low inter-individual variation among dominant foundresses, and this group separated from the other four groups (Figure S2A). For the Dufour’s gland, 11 of 41 compounds differed significantly across the groups. LDA showed large overlap between the two worker groups, and some modest separation among the other three groups, all of which are members of the reproductive caste (Figure S2B). For the sternal gland, only 3 of the 44 compounds showed significant differences across groups. The LDA resulted in four distinct groupings: dominant foundresses, subordinate foundresses, dominant workers/queens, and subordinate workers (Figure S2C).

The chemical profiles of the three exocrine glands- mandibular, Dufour’s, and sternal- did not clearly correlate with dominance status. Each gland showed unique patterns of abundance of numerous different unidentified compounds (Figure S2 A-C). In the mandibular gland, there was a striking separation between dominant foundresses and all other groups, with extremely low variation in chemical profiles in dominant foundresses (Figure S2B). Perhaps there are a small number of compounds produced in the mandibular glands of dominant foundresses that serve an important role in nest marking, dominance, or another unknown function. In the Dufour’s glands, overall chemical profiles may reflect caste, mating status, or egg-laying state of females. Previous studies have shown the Dufour’s gland, which is located in the vicinity of the sting apparatus, may be used in egg-marking in several other Hymenoptera [[6](#_ENREF_6)]. In the sternal glands, overall chemical profiles showed separation between dominant and subordinate foundresses, and both of these were distinct from the profiles of queens and workers. Overall, the chemical profiles of these three glands suggest some compounds (as yet unidentified) differ greatly between dominant and subordinate foundresses, potentially tracking differences in fertility and/or dominance status. However, there were fewer differences between queens and workers, and no evidence of glandular secretions consistently associated with dominance status in both foundresses and queens/workers. Overall, the chemical profiles described here represent a first step in the identification of compounds related to social behavior in *P. metricus*.

**Figure S1**

Each wasp collected was subjected to several dissections, and the body parts of each wasp were used in numerous subsequent analyses. RNA= RNA extractions and microarrays, Chem=chemical analysis using GC and GC-MS, DNA= DNA extractions and microsatellite analysis, WW= wing wear noted to assess foraging experience, Size= ovary size score was noted, Mg= mandibular gland, Dg= Dufour’s gland, Sg= sternal glands. Wasp drawing from Louise Kulzer’s website http://crawford.tardigrade.net/bugs/BugofMonth16.html.

**Figure S2**

Linear discriminant analysis (LDA) of chemical profile data, showing graphs based on values of the two major linear discriminants, derived from quantities of **A)** unknown compounds extracted from mandibular glands **B)** unknown compounds extracted from Dufour’s glands and **C)** unknown compounds extracted from sternal glands. DF= dominant foundress, SF= subordinate foundress, DW= dominant worker, SW= subordinate worker, Q= queen. Numbers in parentheses indicate the number of compounds found to differ significantly across the five groups (in red) over the total number of compounds quantified.

**Figure S3**

A summary of brain gene expression patterns in the five groups (DF= dominant foundress, SF= subordinate foundress, DW= dominant worker, SW= subordinate worker, Q= queen). **A)** Hierarchical clustering with heatmap showing the diversity of expression patterns in each group. **B)** Principal components analysis showing the overall trend in expression pattern for each principal component (PC). **C)** Heatmap distance matrix and dendrogram showing the number of differentially expressed transcripts between each group.

**Figure S4**

A summary of ovary gene expression patterns in the five groups (DF= dominant foundress, SF= subordinate foundress, DW= dominant worker, SW= subordinate worker, Q= queen). **A)** Hierarchical clustering with heatmap showing the diversity of expression patterns in each group. **B)** Principal components analysis showing the overall trend in expression pattern for each principal component (PC). **C)** Heatmap distance matrix and dendrogram showing the number of differentially expressed transcripts between each group.

**Figure S5**

Comparison of real time qRT-PCR data from a previous study (Toth et al. 2007) and microarray data from this study for one gene, *Vg* (contig number cn906 from *P. metricus* transcriptome assembly, Toth et al. 2010). Data from each study were converted to fold change to make the units comparable; this was accomplished by arbitrarily setting the worker expression value to 1 and representing the queen expression value as a fold difference compared to worker. ** indicates a highly significant difference for both the qRT-PCR data (two tailed t-test, p=0.006) and the array data (t_35_= 5.32, p=0.002).

**Additional File 2**

Complete data for 6 microsatellite loci for all nests. Putative ID (DF= dominant foundress, SF= subordinate foundress, DW= dominant worker, SW= subordinate worker, Q= queen) is based on ovary dissections as described in the main text. Based on their genotypes, individuals were grouped into matrilines with founding generation matrilines represented by letters and worker generation matrilines represented by numbers. Based on the matriline reconstructions as well as patterns of allele sharing between individuals, the reconstructed relationship of each individual to their co-foundresses (Founding Phase nests) or to the queen (Worker Phase nests) are indicated. For some individuals, not enough microsatellite information was available to reliably determine relationships. These are designated as “Undetermined”.

**Additional File 3**

“Contig Lists” Tab: Complete list of brain differentially expressed transcripts. The first column “All DE” lists all differentially expressed transcripts. The subsequent columns list transcripts that were significantly different in post-hoc contrasts (DF= dominant foundress, SF= subordinate foundress, DW= dominant worker, SW= subordinate worker, Q= queen). The last two columns give lists of “Dominance-Associated” and “Caste-Associated” transcripts as described in the main text. “Overlaps” Tab: The first ten columns contain lists of overlapping genes from cross-species comparisons presented in Table 1. These are listed as “Overlap 1” through “Overlap 10” and the 11^th^ column specifies what each overlap refers to.

**Additional File 4**

Complete list of ovary differentially expressed transcripts. The first column “All DE” lists all differentially expressed transcripts. The subsequent columns list transcripts that were significantly different in post-hoc contrasts (DF= dominant foundress, SF= subordinate foundress, DW= dominant worker, SW= subordinate worker, Q= queen). The last two columns give lists of “Dominance-Associated” and “Caste-Associated” transcripts as described in the main text.

**References**

1. Strassmann JE: **Selective altruism towards closer over more distant relatives in colonies of the primitively social wasp, *Polistes***. In: *Natural History and Evolution of Paper-wasps.* Edited by Turillazzi S, West-Eberhard MJ. New York: Oxford University Press; 1996.

2. Strassmann JE, Barefield K, Solis CR, Hughes CR, Queller DC: **Trinucleotide microsatellite loci for a social wasp, *Polistes***. *Molecular Ecology* 1997, **6**(1):97-100.

3. Henshaw MT, Robson SKA, Crozier RH: **Queen number, queen cycling and queen loss: the evolution of complex multiple queen societies in the social wasp genus *Ropalidia***. *Behav Ecol Sociobiol* 2004, **55**(5):469-476.

4. Goodnight KF, Queller DC: **Computer software for performing likelihood tests of pedigree relationship using genetic markers**. *Molecular Ecology* 1999, **8**(7):1231-1234.

5. Zanette LR, Field J: **Genetic relatedness in early associations of *Polistes dominulus*: from related to unrelated helpers**. *Molecular Ecology* 2008, **17**(11):2590-2597.

6. Dani FR, Fratini S, Turillazzi S: **Behavioural evidence for the involvement of Dufour's gland secretion in nestmate recognition in the social wasp *Polistes dominulus* (Hymenoptera: Vespidae)**. *Behav Ecol Sociobiol* 1996, **38**(5):311-319.
